# Granulomatous Hepatitis Secondary to *Histoplasma* Infection after Treatment with Infliximab

**DOI:** 10.1155/2013/807537

**Published:** 2013-12-14

**Authors:** L. Barrera, J. Álvarez, M. Tapias, V. Idrovo, R. López

**Affiliations:** ^1^Pathology and Clinical Laboratory Department, Fundación Santa Fe de Bogotá Teaching Hospital, Colombia, Calle 119, No. 7-75, Bogotá, Colombia; ^2^Universidad de los Andes, School of Medicine, Cra 1, No. 18A-12, Bogotá, Colombia; ^3^Gastroenterology Department, Fundación Santa Fe de Bogotá Teaching Hospital, Calle 119, No. 7-75, Bogotá, Colombia

## Abstract

Classical presentation of *Histoplasma* infection includes fever and respiratory symptoms. Opportunistic microorganisms must be suspected on immunocompromised patients who develop bizarre symptoms. We present a case of a female patient with rheumatoid arthritis who received treatment with Infliximab during one and a half year; she developed granulomatous hepatitis secondary to *Histoplasma* infection. The patient was admitted with acute hepatitis and thrombocytopenic coagulopathy. A liver biopsy was performed revealing granulomatous hepatitis, microvesicular steatosis, isolated apoptotic cells, and parenchyma microabscesses. PAS and Gömöri stains revealed areas with mycotic microorganisms morphologically compatible with *Histoplasma* spp. and confirmed by culture.

## 1. Introduction

*Histoplasma capsulatum* (HC) is a dimorphic fungus classified as an opportunistic microorganism; it can affect patients treated with infliximab, which is a monoclonal antibody against tumor necrosis factor alpha (TNF*α*). On the literature we found only 13 reported cases of *Histoplasma* infection (HI) in patients treated with anti-TNF*α* therapy (9 with Infliximab and 4 with Etanercept) [1–7]. The use of these drugs has been associated with increase in the frequency of granulomatous infections [2]. However we present the first case of granulomatous hepatitis (GH) associated with HI in a patient with Rheumatoid Arthritis (RA) treated with Infliximab.

Granulomas are found in 2 to 35% of liver biopsies and up to 30% of these cases remain idiopathic biopsies [8–10]. Its etiology is diverse and is not established in almost half of the cases [11]; therefore histological evaluation of granulomas on liver specimens necessarily needs clinical correlation and the use of special stains for microorganisms including Ziehl-Neelsen, Periodic acid-Schiff (PAS), Grocott's Methenamine Silver Stain (or Gömöri), Giemsa, and Mucicarmine. 

*Case*. A 58-year-old female patient was admitted with previous extrainstitutional one-week hospitalization due to 3 weeks of multiple emesis episodes, weakness, progressive jaundice, and abdominal distention, associated with choluria, acholia, and hyporexia. Suspicious biliary tract obstruction was made and submitted reports informed; abdominal Magnetic Resonance Imaging (MRI) and Retrograde Endoscopic Cholangiography both with evidence of hepatomegaly with normal biliary tree, fatty change, and perihepatic fluid scan, with no focal lesions nor vascular thrombosis, Abdominal Computed Axial tomography (CT) with hepatosplenomegaly (Images not available). Obstruction was ruled out, and because no improvement was achieved, the patient was referred to our institution.

She reported a 1-year diagnosis of RA treated with Infliximab (3 mgr/Kg each two months), Prednisolone (2.5 mgr once a day), Methotrexate (10 mgr once a day), Folic Acid (1 mgr each day), Acetaminophen (500 mgr once a day), and Diclofenac (15 mgr if required). Family history included gastric carcinoma on her father. Physical examination revealed jaundice, no skin lesions, tachycardia, nonpainful hepatomegaly, no ascites, and grade II edema on lower extremities.

Initial laboratory tests demonstrated leukocytes 5.2 ∗ 10^3^/UL (5–10 ∗ 10^3^/UL) with neutrophilia 2.8 (1.4–6.5 ∗ 10^3^/UL), anemia (hemoglobin 11.8 g/L (12–16 gr/dL), hematocrit 34.2% (45–54%)), thrombocytopenia (platelets 59.000 UL (150.000–450.000 UL)), partial thromboplastin time (PTT) 74.70 sec (control 28.8), Prothrombin time (PT) 24.8 sec (control 10.9), INR 2.47, blood urea nitrogen (BUN) 11.6 mgr/dL (6–20 mgr/dL), alkaline phosphatase 376 U/L (32–91 U/L), total serum bilirubin 15.02 mg/dL, direct bilirubin 9.77 mg/dL (6–20 mg/dL), Indirect bilirubin 5.25 mg/dL, aspartate aminotransferase (AST) 581 U/L (15–41 U/L), alanine aminotransferase (ALT) 239 U/L (14–54 U/L). Viral hepatitis serology determined by chemiluminescence; A hepatitis: IgG 6.05 (Reactive), IgM 0.6 (Non-reactive) and B hepatitis surface antigen 0.30 (Non-reactive), IgM antibody 0.06 (Non-reactive). Adrenal function studies were not performed. Diagnosis of multiorganic dysfunction with hematologic component and jaundice of unknown etiology was made.

Studies included liver biopsy (see Section 3) that confirmed the diagnosis of GH and HI. Corticosteroid therapy was discontinued and coagulopathy correction was made through transfusions of blood products. New radiological studies including chest radiography showed interstitial reticulonodular lung alveolar infiltrates, mainly in the right base and bilateral pleural effusions. Abdominal ultrasound (US) showed fatty liver change, scant ascitic fluid, and a normal biliary tree. A contrast abdominal CT was not performed due critical condition.

During hospitalization, she developed respiratory distress, hypotension, and hypoglycemia. Antibiotic management included Meropenem (2 gr three times a day), Amikacin (1000 mgr single dose), Clarithromycin (500 mgr twice a day), and antifungal drug Amphotericin B (5 mg/Kg each day). Patient was transferred to the intensive care unit, but despite the multidisciplinary advance treatment she developed rapidly progressive deterioration with multiorganic failure. Pulmonary HI could not be confirmed through fibrobronchoscopy nor lung biopsy due to serious condition. Finally she required ventilatory support and vasopressor support with norepinephrine (0.6 mcg/Kg/min) and vasopressin (4 UI/H). 72 hours upon her arrival, the patient presented cardio respiratory failure and died.

## 2. Materials and Methods

Liver biopsy (Trucut) obtained 1 fragment processed according to our institutional guides where multiple cut sections (4 um) stained with H&E were generated. Additionally Masson's trichrome, Gram, Mucicarmine, Gömöri trichrome, Ziehl-Neelsen, Reticuline, and Periodic acid-Schiff (PAS) with and without diastase digestion staining were performed.

## 3. Microscopic Examination

**Figure 1:**
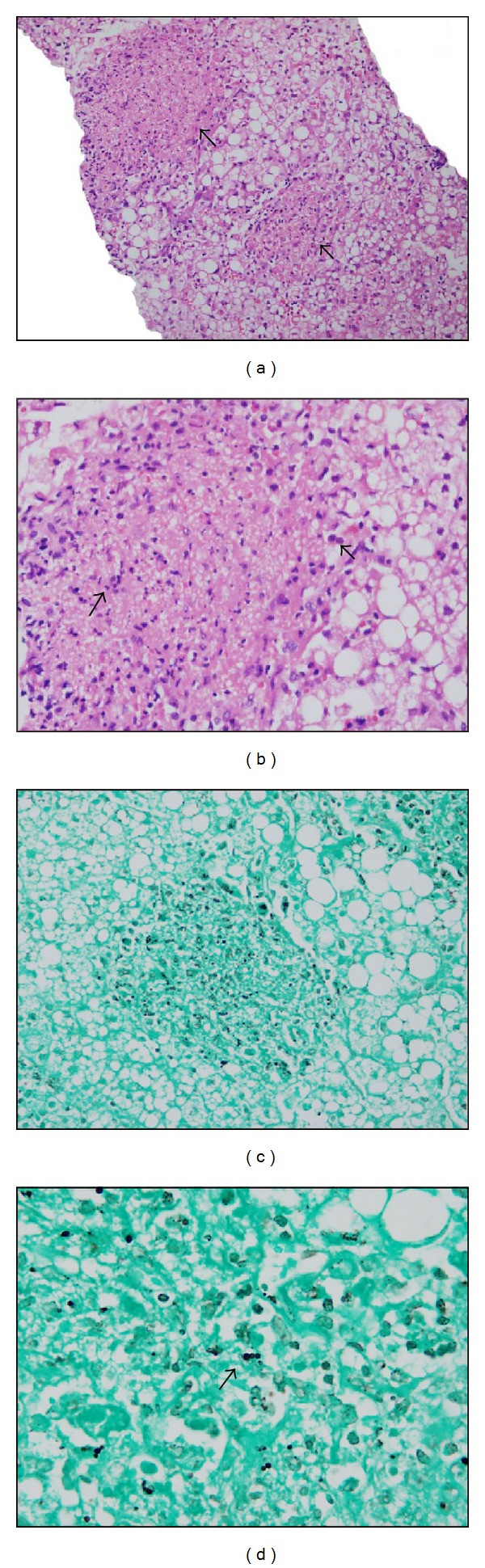
(a) HE ×20, presenting two granulomas (arrows) and (b) HE ×40, showing inflammatory infiltrate and clusters forming numerous microabscesses, abundant polymorphonuclear neutrophils, cellular debris, histiocytes, epithelioid cells, and necrosis. (c) Gömöri ×20 and (d) Gömöri ×40, revealing blastoconidia formations (arrows), morphologically consistent with *Histoplasma*.

Hepatic parenchyma with abnormal architecture had 15 portal tracts, and showed presence of noncaseating granulomas localized in both parenchyma and portal tracts associated with acute inflammatory infiltrate and clusters forming numerous microabscesses (Figure 1(a)), abundant polymorphonuclear neutrophils, cellular debris, histiocytes, epithelioid cells, and necrosis (Figure 1(b)). Neither caseation necrosis nor giant cells were identified. Rest of the parenchyma showed ballooned hepatocytes with severe microvesicular steatosis change in almost 100% of hepatocytes. Special stains Gömöri and PAS identified abundant blastoconidia formations, morphologically consistent with *Histoplasma* (Figures 1(c) and 1(d)), some with intracellular location confirmed with crop. Study was negative for Mucicarmine. Ziehl-Neelsen and Gram stains did not showed any acid-fast bacilli. Final diagnosis was acute GH with multiple microabscesses formation and presence of mycotic microorganisms compatible with HI.

## 4. Discussion

Histoplasmosis caused by HC is found worldwide. In the United States of America (USA) it is the most common systemic mycosis [12] and is recognized as an important reason for respiratory infections in endemic areas, particularly temperate regions in USA and in South America. In this scene HI has to be considered in every immunosuppressed patient, especially when, nowadays, more than 400 000 patients with RA, inflammatory bowel disease, psoriatic arthritis, and ankylosing spondylitis are treated with anti-TNF*α* therapy and since its approval by FDA worries related to its safety appeared, mainly related to reactivation of granulomatous diseases (tuberculosis) [2]. However it is not clear if the latent primary infection represents risk of endogenous reactivation after the use of anti-TNF*α* [12].

In this case report, our patient received during one and a half year anti-TNF*α* concomitant with prednisone because of its immune disease. A strict follow-up of any suspicious signs and symptoms related with the immune condition including the related thrombocytopenic coagulopathy were carefully analyzed. In this way we overemphasize the importance of clinical surveillance (signs and symptoms) combined with imaging and clinical laboratory information including histopathology analysis [13, 14]. However our case had unusual presentation because gastrointestinal histoplasmosis rarely presents together with fever and lung involvement [15]. Pathology was definitive ratifying granulomatous infection on liver, later confirmed by culture.

Immune recovery syndrome must be considered in immunosuppressed patients with an overwhelming inflammatory response. It consists on paradoxical clinical worsening due to Th1 response, including pathogen specific interferon-*γ* and nonspecific TNF-*α* [16]. This response has been described in patients with tuberculosis after discontinuation of TNF-*α* blockers [17]. In this way some studies had shown that occurrence of immune recovery syndrome after stopping TNF blockers is considered a potential cause for clinical deterioration [16]. In our case, progression of histoplasmosis could have accounted for the clinical worsening. These findings could indicate progression of histoplasmosis. An alternative possibility is that they represent “unmasking” of histoplasmosis at the nadir of the TNF blocker effect, as described in patients with AIDS after they start antiretroviral therapy [18].

In this context, with the purpose of preventing fungal infections while anti-TNF*α* therapy, considerations for screening and prevention must include anti-TNF*α* therapy risk of fungal infection, close followup during the first 3 months, suspicion of fungal infection when fever is presented, and avoiding high-risk exposures (cave exploring and bird roosts) and patients from *Coccidioides* endemic areas should have titers checked prior to initiation of anti-TNF therapy [2]. Nevertheless, the exact mechanism by which anti-TNF-a therapy causes reactivation of HC needs further investigation; in this way, some investigations revealed that anti-TNF*α* seems to be a central mediator of protective immunity in HC infections [19].

It is well known that primary infection with HC is usually subclinical or self-limited but may present as a fulminant infection in immunocompromised patients [20]. Its incidence is major after Infliximab therapy [21] and infection can be reported during the first week to 11 months under this treatment; models of explanation include faults on TH1 arm of cellular immunity to HC induced by Infliximab [21, 22]. Likewise Murine models showed that HI was reactivated following depletion of CD4 and CD8 lymphocytes [19].

## 5. Conclusion

This case report displays that although HI was not previously confirmed, its development could be explained from the existing evidence related to anti-TNF*α* therapy where reactivation of Histoplasmosis has been described. By the same, in spite of the exact mechanism by which anti-TNF*α* therapy causes reactivation of HI, anti-TNF*α* has a central role on HI apparently because of faults on TH1 arm of cellular immunity [23]. Prospective studies are needed to accurately assess the risk of fungal infections during treatment with anti-TNF*α* [2]. For the moment, *Histoplasma* antigen detection in urine and serum, and histopathological identification of HC in tissues are mandatory to establish an early diagnosis of HI in immunocompromised patients [20].

## References

[1] Hansen RA, Gartlehner G, Powell GE, Sandler RS (2007). Serious adverse events with infliximab: analysis of spontaneously reported adverse events. *Clinical Gastroenterology and Hepatology*.

[2] Desai SB, Furst DE (2006). Problems encountered during anti-tumour necrosis factor therapy. *Best Practice and Research*.

[3] Keystone EC (2004). The utility of tumour necrosis factor blockade in orphan diseases. *Annals of the Rheumatic Diseases*.

[4] Lizardi-Cervera J, Soto Ramírez LE, Poo JL, Uribe M (2005). Hepatobiliary diseases in patients with human immunodeficiency virus (HIV) treated with non highly active anti-retroviral therapy: frequency and clinical manifestations. *Annals of Hepatology*.

[5] Marie I, Guglielmino E (2010). Non tuberculous anti-TNF associated opportunistic infections. *La Revue de Médecine Interne*.

[6] Bourrtessier J, Fortin C, Belisle A, Desmarais E, Choquette D, Sencal JL (2009). Disseminated Histoplasma capsulatum infection presenting with panniculitis and focal myositis in rheumatoid arthritis treated with etanercept. *Scandinavian Journal of Rheumatology*.

[7] Jain VV, Evans T, Peterson MW (2006). Reactivation histoplasmosis after treatment with anti-tumor necrosis factor α in a patient from a nonendemic area. *Respiratory Medicine*.

[8] Rubin E (1965). Interpretation of the liver biopsy. Diagnostic criteria. *Gastroenterology*.

[9] Klatskin G (1976). Hepatic granulomata: problems in interpretation. *Annals of the New York Academy of Sciences*.

[10] Guckian JC, Perry JE (1966). Granulomatous hepatitis. An analysis of 63 cases and review of the literature. *Annals of Internal Medicine*.

[11] Hussain N, Feld JJ, Kleiner DE (2007). Hepatic abnormalities in patients with chronic granulomatous disease. *Hepatology*.

[12] Yusuf H, Craig GT, Allan D (1979). Disseminated histoplasmosis presenting with oral lesions. Report of a case. *British Journal of Oral Surgery*.

[13] Lee J-H, Slifman NR, Gershon SK (2002). Life-threatening histoplasmosis complicating immunotherapy with tumor necrosis factor α antagonists infliximab and etanercept. *Arthritis and Rheumatism*.

[14] Wood KL, Hage CA, Knox KS (2003). Histoplasmosis after treatment with anti-tumor necrosis factor-α therapy. *American Journal of Respiratory and Critical Care Medicine*.

[15] Bodily K, Perfect JR, Procop G, Washington MK, Affronti J (1996). Small intestinal histoplasmosis: successful treatment with itraconazole in an immunocompetent host. *Gastrointestinal Endoscopy*.

[16] Hage CA, Bowyer S, Tarvin SE, Helper D, Kleiman MB, Wheat LJ (2010). Recognition, diagnosis, and treatment of histoplasmosis complicating tumor necrosis factor blocker therapy. *Clinical Infectious Diseases*.

[17] Belknap R, Reves R, Burman W (2005). Immune reconstitution to Mycobacterium tuberculosis after discontinuing infliximab. *International Journal of Tuberculosis and Lung Disease*.

[18] Nacher M, Sarazin F, El Guedj M (2006). Increased incidence of disseminated histoplasmosis following highly active antiretroviral therapy initiation. *Journal of Acquired Immune Deficiency Syndromes*.

[19] Durkin M, Kohler S, Schnizlein-Bick C (2001). Chronic infection and reactivation in a pulmonary challenge model of histoplasmosis. *Journal of Infectious Diseases*.

[20] Nakelchik M, Mangino JE (2002). Reactivation of histoplasmosis after treatment with infliximab. *American Journal of Medicine*.

[21] Zhang Z, Correa H, Bégué RE (2002). Tuberculosis and treatment with infliximab. *The New England Journal of Medicine*.

[22] Giles JT, Bathon JM (2004). Serious infections associated with anticytokine therapies in the rheumatic diseases. *Journal of Intensive Care Medicine*.

[23] Wallis RS, Broder MS, Wong JY, Hanson ME, Beenhouwer DO (2004). Granulomatous infectious diseases associated with tumor necrosis factor antagonists. *Clinical Infectious Diseases*.

